# Perioperative chemotherapy use and related outcomes in muscle‐invasive bladder cancer in Australia

**DOI:** 10.1002/bco2.70083

**Published:** 2025-09-15

**Authors:** Alison Hiong, James Lynam, Andrew Weickhardt, Shirley Wong, Shomik Sengupta, Paul Manohar, Lih‐Ming Wong, Philip Dundee, Nathan Lawrentschuk, Alison Y. Zhang, Angelyn Anton, Ajay Raghunath, Peter Gibbs, Ben Tran

**Affiliations:** ^1^ Department of Medical Oncology Alfred Health Melbourne Australia; ^2^ School of Translational Medicine Monash University Australia; ^3^ College of Health, Medicine and Wellbeing The University of Newcastle Australia; ^4^ Department of Medical Oncology Calvary Mater Newcastle Waratah Australia; ^5^ Department of Medical Oncology Austin Health Heidelberg Australia; ^6^ Department of Medical Oncology Western Health Australia; ^7^ Department of Urology Eastern Health Box Hill Australia; ^8^ Eastern Health Clinical School Monash University Australia; ^9^ Department of Urology Monash Health Australia; ^10^ Department of Urology St Vincent's Hospital Melbourne Fitzroy Australia; ^11^ Department of Surgery The Royal Melbourne Hospital Parkville Australia; ^12^ Deparmtent of Medical Oncology Chris O'Brien Lifehouse Camperdown Australia; ^13^ Department of Medical Oncology Macquarie University Hospital, Macquarie University Australia; ^14^ Department of Cancer Services Eastern Health Box Hill Australia; ^15^ The Walter and Eliza Hall Institute of Medical Research Parkville Australia; ^16^ Department of Medical Oncology Peter MacCallum Cancer Centre Melbourne Australia

**Keywords:** adjuvant chemotherapy, bladder cancer, immunotherapy, neoadjuvant chemotherapy, real‐world evidence, registry

## Abstract

**Objectives:**

To explore Australian data on perioperative chemotherapy use and associated outcomes in muscle‐invasive bladder cancer (MIBC).

**Subjects and Methods:**

An observational study of patients with MIBC treated with neoadjuvant chemotherapy, adjuvant chemotherapy or surgery alone was conducted using data from BLADDA, a multicentre Australian urothelial cancer registry. Pathological response to neoadjuvant chemotherapy and its effect on event‐free survival (EFS) and overall survival (OS) were determined. EFS and OS in patients who underwent neoadjuvant chemotherapy, adjuvant chemotherapy or surgery alone were compared using univariate and multivariable proportional hazards regression.

**Results:**

From 2018 to 2024, 259 patients enrolled in the BLADDA registry met inclusion criteria, of which 45% received neoadjuvant chemotherapy, 23% received adjuvant chemotherapy, 1.2% received both neoadjuvant and adjuvant chemotherapy and 31% underwent surgery only. The proportion of patients treated with neoadjuvant chemotherapy increased over time. A total of 21 of 67 (31%) evaluable subjects achieved a pathological complete response, which was associated with improved EFS and OS. Excluding patients who received both neoadjuvant and adjuvant chemotherapy, the EFS hazard ratio (HR) was 0.43 (95% confidence interval [CI] 0.29–0.65, p < 0.001) for neoadjuvant chemotherapy and 0.59 (95% CI 0.38–0.94, p = 0.03) for adjuvant chemotherapy compared to surgery alone. Neoadjuvant chemotherapy was associated with prolonged OS in the univariate analysis (HR 0.43, 95% CI 0.26–0.73, p = 0.002) but not in the multivariable analysis (HR 0.59, 95% CI 0.32–1.08, p = 0.09). OS was not improved with adjuvant chemotherapy (unadjusted HR 0.76, 95% CI 0.44–1.31, p = 0.3; adjusted HR 0.86, 95% CI 0.46–1.60, p = 0.6).

**Conclusion:**

Neoadjuvant chemotherapy use for MIBC in Australia has increased over the past decade, but it remains underutilised. This has important implications as perioperative chemo‐immunotherapy emerges as a standard of care. Although a clear impact on survival in the overall population was not observed, this was potentially due to the limited sample size.

## 1 INTRODUCTION

The gold standard management of muscle‐invasive bladder cancer (MIBC) consists of neoadjuvant cisplatin‐based chemotherapy followed by radical cystectomy.^1^, ^2^ Whereas the evidence for adjuvant chemotherapy in MIBC is more equivocal,^3^, ^4^, ^5^ the neoadjuvant approach is backed by data from randomised clinical trials and meta‐analyses demonstrating improved survival when neoadjuvant chemotherapy is combined with surgery, compared to surgery alone.^6^, ^7^, ^8^ Neoadjuvant chemotherapy regimens validated in prospective clinical trials include MVAC (methotrexate, vinblastine, doxorubicin and cisplatin),^6^ cisplatin‐gemcitabine and dose‐dense MVAC (dd‐MVAC), which is associated with longer overall survival and higher pathological response rates than cisplatin‐gemcitabine, but at the expense of more grade ≥3 toxicity.^9^, ^10^ Further to this, recent data supporting the addition of adjuvant nivolumab^11^ and perioperative durvalumab^12^ to platinum neoadjuvant chemotherapy have emerged, leading to the incorporation of immunotherapy into the modern MIBC treatment paradigm.

Despite the established benefit of neoadjuvant therapies in clinical trials, international series have reported limited real‐world uptake,^3^, ^13^ and anecdotally, utilisation rates of neoadjuvant chemotherapy in Australia are low. This may be explained by the frequency of contraindications to cisplatin‐based chemotherapy in real‐world populations, such as renal dysfunction or poor performance status. Other possible reasons for this include clinician uncertainty regarding the superiority of neoadjuvant therapy over adjuvant therapy and a lack of clinician confidence in the external validity of clinical trial data.

Therefore, to describe the MIBC treatment landscape in Australia, evaluate the utilisation of neoadjuvant and adjuvant chemotherapy and explore its real‐world outcomes, we analysed data from BLADDA, a urothelial cancer registry. We also examined the potential implications of current and recent historical treatment trends on future utilisation of perioperative immunotherapy as a standard of care.

## 2 SUBJECTS AND METHODS

BLADDA is a prospective multicentre registry of patients with muscle‐invasive or metastatic urothelial cancer. Subjects are identified by treating clinicians at participating sites and are eligible for inclusion if aged 18 years or above with a diagnosis of muscle‐invasive or metastatic urothelial carcinoma. Collection of data commenced on 23 January 2018. The BLADDA registry allows subjects at any timepoint of muscle‐invasive or metastatic urothelial cancer treatment to enrol and prospectively records data regarding patient demographics, diagnosis, treatment and outcomes. The registry also permits retrospective collection of data regarding any previous bladder cancer treatment received before enrolment. At the time of data cut‐off, all participating sites were located in metropolitan areas of Victoria and New South Wales, Australia.

In this study, we evaluated patients from the BLADDA database with T2–4, N0–3 and M0 bladder cancer, according to the eighth edition of the American Joint Committee on Cancer staging system,^14^ who had undergone surgical resection or had commenced neoadjuvant chemotherapy with a plan to undergo definitive surgery. Subjects were classified based on the treatment they received: neoadjuvant chemotherapy followed by surgery (without adjuvant chemotherapy), surgery followed by adjuvant chemotherapy (without neoadjuvant chemotherapy), surgery with both neoadjuvant and adjuvant chemotherapy, or surgery only. Patients who commenced neoadjuvant chemotherapy but had not (yet) undergone surgery were included in the neoadjuvant chemotherapy population so as not to exclude any patients who developed progressive disease during neoadjuvant therapy or were unable to proceed to surgery for any reason. Differences in baseline characteristics between the treatment groups were evaluated using Fisher's exact test for categorical variables and the Kruskal‐Wallis test for continuous variables.

Pathological T stage and N stage at the time of definitive surgery were recorded. A pathological complete response was defined as the absence of residual invasive carcinoma in the resected primary bladder tumour and all sampled lymph nodes (ypT0 ypN0 or ypTis ypN0).

For each patient, we determined overall survival (OS), defined as the time from the diagnosis of MIBC to death from any cause, and event‐free survival (EFS), defined as the time from MIBC diagnosis to invasive bladder cancer recurrence, disease progression preventing definitive surgery or death from any cause (whichever event occurred first). The Kaplan–Meier method was used to calculate median EFS and OS and their 95% confidence intervals (CIs). Patients who had not died were censored for survival using the date of last review.

EFS and OS in the three main treatment groups (neoadjuvant chemotherapy, adjuvant chemotherapy or surgery alone) were compared using univariate and multivariable Cox proportional hazards regression, generating unadjusted and adjusted hazard ratios (HRs), respectively. In the multivariable survival analyses, the effect of treatment group on EFS and OS was adjusted for sex, age, Eastern Cooperative Oncology Group (ECOG) performance status (0 or 1–2), T stage at diagnosis (T2, T3–4 or TX), N stage at diagnosis (N0, N1 or NX) and the presence of renal impairment (estimated glomerular filtration rate [eGFR] ≥60 ml/min/1.73 m^2^, eGFR <60 ml/min/1.73 m^2^ or eGFR unknown). A cut‐off of 60 ml/min/1.73 m^2^ for eGFR was selected, as cisplatin is typically recommended to be dose‐reduced or avoided below this threshold.^15^ The Breslow method was used to handle subjects with tied survival times. Proportional hazards assumptions were tested using Schoenfeld residuals.

As a supplementary analysis, the Cox regression models were repeated after excluding patients who received non‐cisplatin‐based neoadjuvant chemotherapy regimens. A further exploratory survival analysis was conducted on the subpopulation of subjects with eGFR ≥60 ml/min/1.73 m^2^ in order to exclude individuals who were cisplatin‐ineligible due to inadequate kidney function and thereby limit the potential confounding effect of renal dysfunction.

Patients who received both neoadjuvant and adjuvant chemotherapy were included in analyses relating to pathological response, but excluded from any survival analyses comparing treatment groups due to an insufficient number of subjects in this arm to produce a valid Cox proportional hazards model.

All presented p values are two‐sided. A significance level of 0.05 was used for all analyses. Data analysis was carried out using Stata, version 15.1 (StataCorp, College Station, Texas, United States of America).

This research was conducted in accordance with the principles of the Declaration of Helsinki. Ethical approval for the research was reviewed and granted by the Melbourne Health Human Research Ethics Committee.

## 3 RESULTS

### 3.1 Characteristics of study population

At the time of data cut‐off on 10 January 2024, there were 259 patients in the BLADDA database with MIBC meeting eligibility criteria, with a median follow‐up time of 23.7 months. In total, 81 patients (31%) were treated with surgery alone, 116 patients (45%) were treated with neoadjuvant chemotherapy, 59 patients (23%) were treated with adjuvant chemotherapy and 3 patients (1.2%) received both neoadjuvant and adjuvant chemotherapy. The neoadjuvant chemotherapy group included 30 patients (12%) who had received neoadjuvant chemotherapy and were planned for surgical resection but had not (yet) undergone surgery.

Characteristics of the study population are displayed in Table 1. The median age was 69 years (range 43–88 years). 76% of the population was male. Statistically significant associations were observed between choice of treatment and age, T stage and N stage. The most used neoadjuvant chemotherapy regimens were cisplatin and gemcitabine (43%), dd‐MVAC (29%) and MVAC (15%). In the adjuvant setting, the most common regimens were cisplatin and gemcitabine (61%) and carboplatin and gemcitabine (23%).

**TABLE 1:** Characteristics of study population.

	Neoadjuvant chemotherapy (n = 116)	Adjuvant chemotherapy (n = 59)	Surgery only (n = 81)	Neoadjuvant and adjuvant chemotherapy (n = 3)	p*
Age (years) – median (range)	68 (44–80)	66 (46–83)	73 (43–88)	70 (55–71)	0.001
Sex – n (%)					0.220
Female	24 (21)	11 (19)	25 (31)	1 (33)	
Male	92 (79)	48 (81)	56 (69)	2 (67)	
ECOG – n (%)					0.8
0	73 (63)	37 (63)	51 (63)	2 (67)	
1	23 (20)	12 (20)	16 (20)	0 (0)	
2	1 (0.86)	0 (0)	3 (3.7)	0 (0)	
Unknown	19 (16)	10 (17)	11 (14)	1 (33)	
T stage – n (%)					<0.001
2	94 (81)	24 (41)	41 (51)	3 (100)	
3	12 (10)	27 (46)	30 (37)	0 (0)	
4	4 (3.4)	6 (10)	6 (7.4)	0 (0)	
X	6 (5.2)	2 (3.4)	4 (4.9)	0 (0)	
Clinical N stage – n (%)					0.02
0	63 (54)	23 (39)	43 (53)	0 (0)	
1	7 (6.0)	10 (17)	7 (8.6)	0 (0)	
2	4 (3.4)	7 (12)	8 (9.9)	0 (0)	
3	2 (1.7)	0 (0)	1 (1.2)	1 (33)	
X	40 (34)	19 (32)	22 (27)	2 (67)	
eGFR – n (%)					<0.001
≥60 ml/min/1.73 m^2^	95 (82)	42 (71)	38 (47)	2 (67)	
<60 ml/min/1.73 m^2^	16 (14)	16 (27)	22 (27)	1 (33)	
Unknown	5 (4.3)	1 (1.7)	21 (26)	0 (0)	
Chemotherapy regimen					
MVAC	17 (15)	4 (6.8)	‐	1 (33)**	
dd‐MVAC	34 (29)	2 (3.4)	‐	1 (33)**	
Cisplatin‐gemcitabine	50 (43)	36 (61)	‐	3 (100)**	
Carboplatin‐gemcitabine	7 (6.0)	13 (22)	‐	1 (33)**	
Carboplatin‐etoposide	2 (1.7)	2 (3.4)	‐	0 (0)	
Cisplatin (monotherapy)	2 (1.7)	0 (0)	‐	0 (0)	
Carboplatin (monotherapy)	1 (0.86)	0 (0)	‐	0 (0)	
Mitomycin‐fluorouracil	2 (1.7)	2 (3.4)	‐	0 (0)	
Unknown	1 (0.86)	0 (0)	‐	0 (0)	

* Calculated using Fisher's exact test for categorical variables and the Kruskal‐Wallis test for continuous variables.

** Patients received different regimens in the neoadjuvant and adjuvant setting (one received neoadjuvant MVAC and adjuvant cisplatin‐gemcitabine, one received neoadjuvant cisplatin‐gemcitabine and adjuvant carboplatin‐gemcitabine and one received neoadjuvant ddMVAC and adjuvant cisplatin‐gemcitabine).

ECOG: Eastern Cooperative Oncology Group; eGFR: estimated glomerular filtration rate; MVAC: methotrexate, vinblastine, doxorubicin, cisplatin; dd‐MVAC: dose‐dense methotrexate, vinblastine, doxorubicin, cisplatin.

**FIGURE 1:**
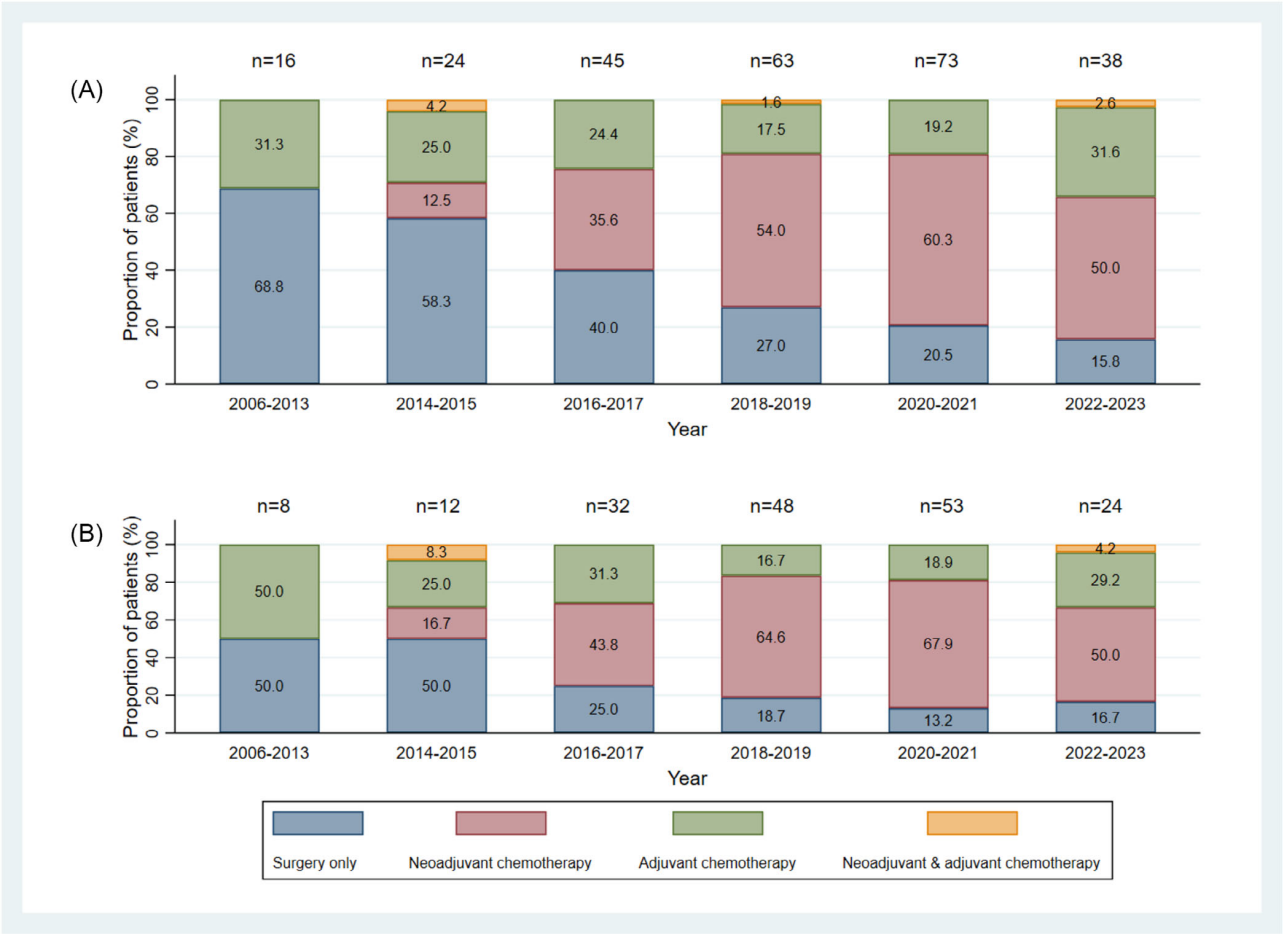
Use of neoadjuvant and adjuvant chemotherapy over time in the total population (a) and in patients with eGFR ≥60 min/1.73 m^2^ (b).

The proportion of patients receiving neoadjuvant chemotherapy increased over time (Figure 1a). Prior to 2014, no patients received neoadjuvant therapy. From the 2018 to 2019 period onwards, neoadjuvant chemotherapy was administered to 57% of patients, but surgery alone and adjuvant chemotherapy remained in use in a minority of patients, including those with eGFR ≥60 ml/min/1.73 m^2^ (Figure 1b).

### 3.2 Pathological response

Pathological staging data at definitive surgery were captured for 162 patients (Table 2), consisting of 67 in the neoadjuvant chemotherapy group (including 3 who received both neoadjuvant and adjuvant chemotherapy), 37 in the adjuvant chemotherapy group and 58 in the surgerly only group.

**TABLE 2:** Pathological stage at definitive surgery.

	Neoadjuvant chemotherapy* (n = 67)	Adjuvant chemotherapy (n = 37)	Surgery only (n = 58)
pT0 pN0	14 (21%)	1 (2.7%)	1 (1.7%)
pT0is pN0	7 (10%)	0 (0%)	1 (1.7%)
pT1 pN0	3 (4.5%)	0 (0%)	2 (3.5%)
pT2–4 pN0	26 (39%)	22 (59%)	35 (60%)
pT0–4 pN1–3	17 (25%)	14 (38%)	19 (33%)

* Includes 3 patients who received both neoadjuvant and adjuvant chemotherapy.

Of the 67 patients treated with neoadjuvant chemotherapy, 31% (n = 21) achieved a pathological complete response and 64% (n = 43) had ypT2–4 or ypN + disease. In contrast, 3.5% (n = 2) of the surgery‐only and 2.7% (n = 1) of the adjuvant chemotherapy groups were adjudicated to have had no residual disease at the time of surgery.

**FIGURE 2:**
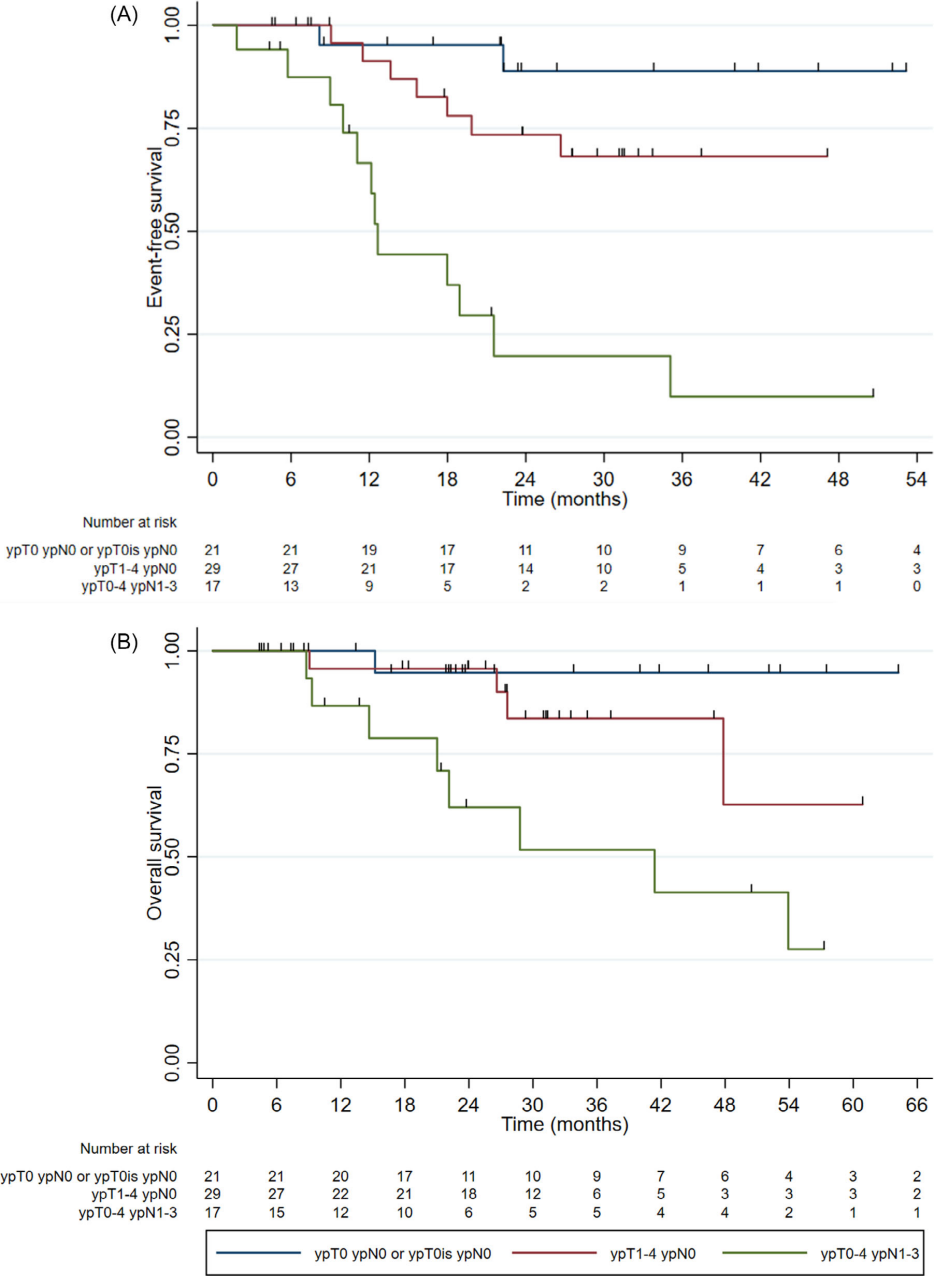
Kaplan–Meier curves for event‐free survival (a) and overall survival (b) according to pathological response in patients treated with neoadjuvant chemotherapy.

In the patients who received neoadjuvant chemotherapy, a pathological complete response was associated with improved EFS and OS. In the case of EFS (Figure 2a), when compared to complete responders, the HR in patients with residual node‐negative disease (ypT1–4 ypN0) was 2.18 (95% CI 0.56–8.45, p = 0.3), while the HR for node‐positive residual disease (ypT0–4 ypN1–3) was 12.0 (95% CI 3.27–44.0, p < 0.001). Compared to complete responders, the mortality HR was 1.80 (95% CI 0.33–9.89, p = 0.5) in patients with residual node‐negative disease and 6.15 (95% CI 1.30–29.02, p = 0.02) in patients with residual node‐positive disease (Figure 2b).

### 3.3 Event‐free survival

**FIGURE 3:**
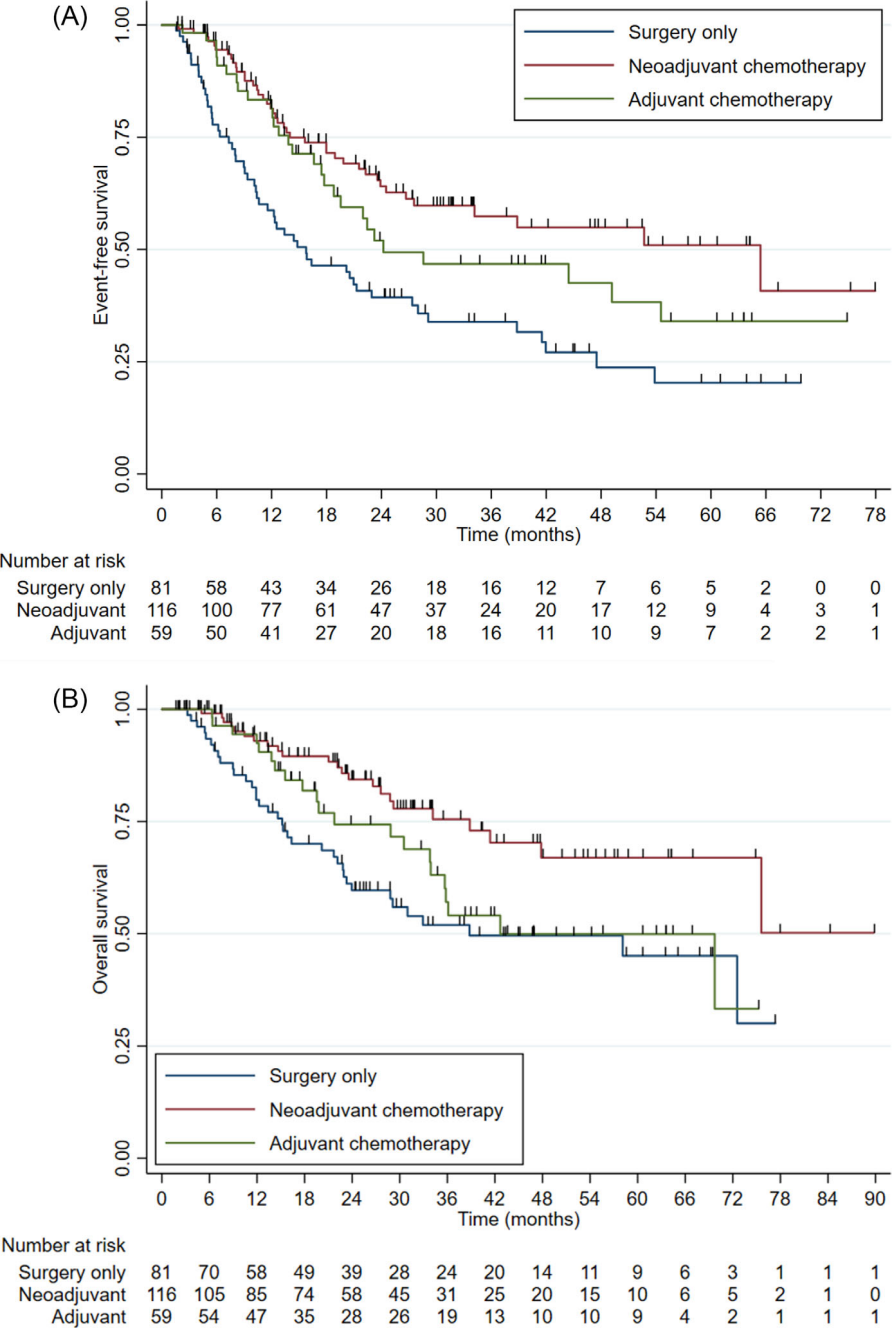
Kaplan–Meier curves for event‐free survival (a) and overall survival (b) according to treatment group.

At the data cut‐off date, 48% (n = 123) of 256 evaluable patients (having excluded those who received both neoadjuvant and adjuvant chemotherapy) had experienced an event, including 65% (n = 53), 35% (n = 41) and 49% (n = 29) of the surgery, neoadjuvant chemotherapy and adjuvant chemotherapy groups, respectively. Median EFS (Figure 3a) was 28.0 months (95% CI 22.0–47.5 months) in the total population, 15.8 months (95% CI 10.4–27.4 months) in the surgery group, 65.4 months (95% CI 27.6 months‐not reached) in the neoadjuvant chemotherapy group and 24.2 months (95% CI 17.7 months‐not reached) in the adjuvant chemotherapy group.

Compared to surgery alone, patients who received either neoadjuvant or adjuvant chemotherapy had longer EFS. The unadjusted HR for neoadjuvant chemotherapy relative to surgery was 0.43 (95% CI 0.29–0.65, p < 0.001) and the unadjusted HR for adjuvant chemotherapy relative to surgery was 0.59 (95% CI 0.38–0.94, p = 0.03).

In the 175 patients with eGFR ≥60 ml/min/1.73 m^2^, neoadjuvant and adjuvant chemotherapy were also associated with improved EFS over surgery alone; the HR was 0.39 (95% CI 0.23–0.66, p = 0.001) in the neoadjuvant chemotherapy group and 0.50 (95% CI 0.27–0.91, p = 0.02) in the adjuvant chemotherapy group.

The multivariable Cox proportional hazards model for EFS (Table 2) also indicated an advantage with the use of either neoadjuvant chemotherapy (adjusted HR 0.54, 95% CI 0.33–0.86, p = 0.01) or adjuvant chemotherapy (adjusted HR 0.49, 95% CI 0.29–0.83, p = 0.008) over surgery alone. Using the same model, the adjusted HR for neoadjuvant chemotherapy expressed relative to adjuvant chemotherapy was 1.10 (95% CI 0.64–1.87, p = 0.7).

### 3.4 Overall survival

The proportion of patients who died was 31% (n = 80) in the total population, 44% (n = 36) among those treated with surgery alone, 20% (n = 23) among those treated with neoadjuvant chemotherapy and 36% (n = 21) among those treated with adjuvant chemotherapy. Median OS was 38.8 months (95% CI 23.3 months‐not reached) in the surgery group and 42.7 months (95% CI 33.8 months‐not reached) in the adjuvant chemotherapy group (Figure 3b). Median OS was not reached in the neoadjuvant chemotherapy group (95% CI 75.6 months‐not reached).

In the univariate analysis, the risk of death was lower in individuals treated with neoadjuvant chemotherapy compared to those who received surgery only, with an unadjusted HR of 0.43 (95% CI 0.26–0.73, p = 0.002). The unadjusted HR for death associated with adjuvant chemotherapy relative to surgery alone was 0.76 (95% CI 0.44–1.31, p = 0.3).

In patients with eGFR ≥60 ml/min/1.73 m^2^, the data remained consistent with neoadjuvant chemotherapy adding benefit, with an unadjusted HR of 0.37 (95% CI 0.18–0.75, p = 0.006). Adjuvant chemotherapy was not associated with improved survival over surgery alone in this subgroup (unadjusted HR 0.81, 95% CI 0.40–1.63, p = 0.5).

The multivariable analysis (Table 3) did not indicate that neoadjuvant chemotherapy reduced the risk of death relative to surgery alone (adjusted HR 0.59, 95% CI 0.32–1.08, p = 0.09). Similarly, adjuvant chemotherapy was not associated with improved OS (adjusted HR 0.86, 95% CI 0.46–1.60, p = 0.6). Expressed relative to adjuvant chemotherapy, the adjusted HR for neoadjuvant chemotherapy was 0.68 (95% CI 0.35–1.32, p = 0.3).

**TABLE 3:** Multivariable Cox proportional hazards models for event‐free survival and overall survival in total population.

	Event‐free survival			Overall survival		
	HR	95% CI	p	HR	95% CI	p
Treatment group						
Surgery only	Reference	‐	‐	Reference	‐	‐
Neoadjuvant chemotherapy	0.54	0.33–0.86	0.01	0.59	0.32–1.08	0.09
Adjuvant chemotherapy	0.49	0.29–0.83	0.008	0.86	0.46–1.60	0.6
Age (years)	1.03	1.01–1.05	0.009	1.03	1.01–1.06	0.02
Sex						
Female	Reference	‐	‐	Reference	‐	‐
Male	0.88	0.57–1.34	0.5	0.85	0.50–1.42	0.5
ECOG performance status						
0	Reference	‐	‐	Reference	‐	‐
1–2	1.11	0.71–1.76	0.6	2.08	1.19–3.61	0.01
T stage						
T2	Reference	‐	‐	Reference	‐	‐
T3–4	1.58	1.03–2.43	0.04	0.96	0.57–1.62	0.9
TX	1.20	0.53–2.75	0.7	1.58	0.61–4.06	0.3
N stage						
Node‐negative	Reference	‐	‐	Reference	‐	‐
Node‐positive	2.57	1.57–4.21	<0.001	3.27	1.81–5.91	<0.001
NX	1.37	0.87–2.15	0.2	1.14	0.65–2.01	0.6
eGFR						
≥60 ml/min/1.73 m^2^	Reference	‐	‐	Reference	‐	‐
<60 ml/min/1.73 m^2^	1.14	0.72–1.81	0.6	1.36	0.80–2.33	0.3

HR: hazard ratio, CI: confidence interval, ECOG: Eastern Cooperative Oncology Group, eGFR: estimated glomerular filtration rate.

When the neoadjuvant chemotherapy group was restricted to patients who received cisplatin‐based regimens, neoadjuvant chemotherapy was associated with significantly better OS compared to surgery alone in both the univariate model, which showed an adjusted HR of 0.39 (95% CI 0.22–0.68, p = 0.001), and multivariable model, which showed an adjusted HR of 0.52 (95% CI 0.28–0.99, p = 0.046).

## 4 DISCUSSION

In this analysis of the BLADDA registry, we demonstrated that neoadjuvant chemotherapy had a beneficial impact in the treatment of MIBC in an Australian, real‐world population. Patients who received neoadjuvant chemotherapy had a lower risk of invasive bladder cancer recurrence and death compared to those who had surgery without chemotherapy. A complete pathological response was achieved in 31% of evaluable patients treated with neoadjuvant chemotherapy, which is comparable to pathological complete response rates reported in prospective clinical trials.^12^ In addition, patients who achieved a complete pathological response to neoadjuvant therapy had longer event‐free survival and overall survival compared to those who had residual node‐positive disease after chemotherapy. Adjuvant chemotherapy was associated with improved event‐free survival but not overall survival.

Acknowledging a non‐randomised population with some imbalances in baseline characteristics and potential imbalances in other prognostic markers that were not captured by the registry data, we attempted to account for potential selection bias using multivariable regression. This corroborated the favourable effect of neoadjuvant chemotherapy on survival in patients who received cisplatin‐containing regimens but did not produce clear evidence of advantage in the overall population. In keeping with this observation, non‐cisplatin‐based regimens do not have well‐established efficacy in the neoadjuvant treatment of MIBC,^16^ and hence international management guidelines advise against their use in this setting.^1^

The proportion of subjects receiving neoadjuvant chemotherapy in BLADDA institutions was 46% over the entire follow‐up period. Notably, there was an increase in utilisation of neoadjuvant chemotherapy across the study period, reflecting incremental changes in clinical practice over time. This finding was consistent with expectation, and aligned with the growth in the body of evidence supporting neoadjuvant therapy during this period. Comparing our results to other regions of the world, a Korean nationwide retrospective study found a neoadjuvant chemotherapy utilisation rate that increased from 6.4% to 12% from 2004 to 2016,^3^ while a study of the National Cancer Database in the United States reported a neoadjuvant chemotherapy utilisation rate of 31% from 2010 to 2016.^13^

From 2020 to 2023, 55% of BLADDA registry patients with eGFR ≥60 ml/min/1.73 m^2^ (a level deemed safe for administration of cisplatin) received neoadjuvant chemotherapy. Such an incomplete uptake of neoadjuvant chemotherapy, even in a subpopulation that had adequate renal function for cisplatin and sufficient performance status to undergo radical cystectomy, may in part be explained by other factors contraindicating platinum chemotherapy, such as hearing impairment, active infection or other comorbidities, or by patients choosing to decline a recommendation of neoadjuvant therapy. These factors were not prospectively captured in the BLADDA registry. Furthermore, the available data did not account for variant histology and its impact on chemotherapy sensitivity, which may have influenced clinicians to omit chemotherapy and potentially contributed to unmeasured confounding in the survival analyses.

It is worth emphasising that the data in this study relate to patients who were treated in the era before immunotherapy was established as a standard component of MIBC management. The arrival of immunotherapy in the MIBC treatment landscape occurred later, propelled by the CheckMate 274 study, which showed that use of adjuvant nivolumab in high‐risk MIBC improved disease‐free survival and overall survival.^11^ The NIAGARA study subsequently demonstrated that adding neoadjuvant and adjuvant durvalumab to four cycles of neoadjuvant cisplatin and gemcitabine led to a higher pathological complete response rate and significantly longer overall survival.^12^

With the advent of immunotherapy as a standard of care in the management of MIBC, omission of platinum neoadjuvant chemotherapy has implications beyond loss of benefit from the chemotherapy itself; it may also lead to a reduction in the magnitude of benefit from perioperative immunotherapy or loss of opportunity to receive immunotherapy altogether in the curative‐intent setting. The CheckMate 274 study population consisted of patients with ypT2‐T4a or ypN + MIBC after neoadjuvant cisplatin and pT3‐T4a or pN + MIBC without prior neoadjuvant cisplatin. Subgroup analysis suggested that the favourable impact of adjuvant nivolumab on overall survival was more evident in patients who had received cisplatin‐containing neoadjuvant therapy.^17^ Accordingly, in Australia, publicly‐subsidised access to adjuvant nivolumab for MIBC is only available to patients who have received platinum‐containing neoadjuvant chemotherapy.^18^ In the BLADDA population, over 60% of patients who received neoadjuvant chemotherapy had ypT2 + and/or ypN + disease at surgery, and therefore, in accordance with CheckMate 274 criteria, would have been eligible for adjuvant nivolumab (in the absence of contraindication to immunotherapy). The addition of adjuvant nivolumab in this subpopulation represents an obvious opportunity to improve survival outcomes, while further gains could be achieved by increasing the proportion of patients treated with neoadjuvant platinum chemotherapy to begin with, enabling subsequent access to immunotherapy.

Similarly, the NIAGARA perioperative chemo‐immunotherapy strategy could improve survival by increasing pathological complete response rates. However, this regimen consists of a cisplatin chemotherapy backbone and is therefore only relevant if neoadjuvant chemotherapy is initiated in the first place. In the BLADDA population, 54% of patients did not receive neoadjuvant chemotherapy and would consequently have forgone the opportunity to be treated with perioperative durvalumab.

Weaknesses of this study include the relatively small sample size, the observational nature of the data and inherent limitations associated with registry data quality for certain data points. While the BLADDA registry covers multiple sites across the two most populous states in Australia, it is not a population‐based registry enrolling consecutive patients and does not have complete coverage of all bladder cancer cases in the participating sites. This particular limitation will be attenuated in the future, as additional sites in other regions of Australia have opened since this study's data cut‐off date, and sites in Asia, including Singapore, Korea and Japan, are in the process of joining BLADDA.

Overall, we demonstrated the association of neoadjuvant chemotherapy with pathological response, event‐free survival and overall survival in Australian patients with MIBC. While the use of neoadjuvant chemotherapy has increased over the past 10 years, our results suggest ongoing underutilisation, highlighting the potential for patient outcomes to be improved through its increased use, particularly in combination with adjuvant or perioperative immunotherapy.

## AUTHOR CONTRIBUTIONS

**Alison Hiong:** Formal analysis; writing—original draft; visualisation. **James Lynam:** Writing—review and editing. **Andrew Weickhardt:** Writing—review and editing. **Shirley Wong:** Writing—review and editing. **Shomik Sengupta:** Writing—review and editing. **Paul Manohar:** Writing—review and editing. **Lih‐Ming Wong:** Writing—review and editing. **Philip Dundee:** Writing—review and editing. **Nathan Lawrentschuk:** Writing—review and editing. **Alison Y. Zhang:** Writing—review and editing. **Angelyn Anton:** Writing—review and editing. **Ajay Raghunath:** Writing—review and editing. **Peter Gibbs:** Writing—review and editing. **Ben Tran:** Conceptualisation; writing—review and editing; supervision; project administration.

## CONFLICT OF INTERESTS STATEMENT

The authors have no relevant conflicts of interest to declare.
